# Macrophage activation syndrome in children with systemic juvenile idiopathic arthritis: a retrospective study on 7 patients

**DOI:** 10.1186/1546-0096-12-S1-P218

**Published:** 2014-09-17

**Authors:** Aida Omercahic Dizdarevic, Senka Mesihovic Dinarevic, Velma Selmanovic, Adisa Cengic

**Affiliations:** 1 Allergology, Rheumatology and clinical immunology, Children hospital, Sarajevo, Bosnia and Herzegovina; 2 Cardiology, University Clinic Sarajevo, Children hospital, Sarajevo, Bosnia and Herzegovina

## Introduction

macrophage activation syndrome (MAS) is life-threatening complication of rheumatic diseases and is most frequent seen in systemic juvenile idiopathic arthritis (sJIA). Prompt recognition and immediate therapy is life saving.

## Objectives

to review clinical and laboratory data of MAS in 7 children with sJIA.

## Methods

clinical and laboratory data of 7 patients with MAS, treated in our hospital from January 2008 to December 2013, were analyzed retrospectively.

## Results

seven children (4 females, 3 males) were studied. Two children had incomplete MAS. The underlying disease was not identified in one child. MAS developed during the course of underlying disease (sJIA) in three children. Clinical manifestations at diagnosis included high persistent fever (7), skin rash (6), hepatosplenomegaly (7), lymphadenopathy (6), hemorrhages (5) and central nervous system dysfunction (6). Laboratory data included: high feritin>10 000 (7), cytopenia (7), abnormal liver function tests (7), hypoalbunaemia (7), hypertriglyceridemia (5), coagulopathy (5), decreased erythrocyte sedimentation rate (5). Macrophage hemophagocytosis were found in 4 bone marrow aspiration. Rota virus was isolated in stool in 3 children. MAS was reccurent in two children (perforin gene done, negative). Six children responded on immunosupressive therapy and are doing well, one child died.

## Conclusion

MAS is rare but serious complication of systemic juvenile idiopathic arthritis in children. It is important to keep in mind suddenly clinical and laboratory disturbanses in children with JIA, to recognise and immediate treat MAS in order to decrease mortality.

## Disclosure of interest

None declared.

